# Blocking neuropilin-2 enhances corneal allograft survival by selectively inhibiting lymphangiogenesis on vascularized beds

**Published:** 2010-11-09

**Authors:** Xian-ling Tang, Jun-feng Sun, Xi-ying Wang, Ling-ling Du, Ping Liu

**Affiliations:** 1 Eye Hospital, First Affiliated Hospital, Harbin Medical University, Harbin, PR China; 2 Department of Cardiovascular Medicine, First Affiliated Hospital, Harbin Medical University, Harbin, PR China

## Abstract

**Purpose:**

To investigate the potential inhibitory effects of RNA interference-mediated knockdown of neuropilin-2 (*NP2*) on inflammation-induced corneal hemangiogenesis and lymphangiogenesis, and whether selective inhibition of lymphangiogenesis on vascularized recipient beds before transplantation improves the graft survival.

**Methods:**

Mouse lymphatic endothelial cells were transfected with the plasmid expressing artificial microRNA (amiRNA) against mouse *NP2*, and the down-regulation of VEGF-C-induced *NP2* expression by *NP2* amiRNA was evaluated by real-time PCR and western blot assays. Next, *NP2* amiRNA or negative control amiRNA was injected intrastromally into BALB/c mouse model of suture-induced corneal neovascularization three days after surgery. Corneas were harvested 1 week after suture placement and the formation of lymphatic and blood vessels as well as the recruitment of macrophage was evaluated by immunohistochemical staining. The neovascularized graft beds treated by *NP2* amiRNA or control then served as recipients of orthotopic corneal transplants, and age-matched C57BL/6 donors were used. Corneal allografts were examined twice a week for 8 weeks, and graft clarity was quantified by means of an opacity score.

**Results:**

VEGF-C-induced *NP2* expression at both mRNA and protein levels was significantly suppressed by *NP2* amiRNA in mouse lymphatic endothelial cells. Intrastromal administration of *NP2* amiRNA reduced corneal lymphangiogenesis by 45% versus control (p=0.015), but corneal hemangiogenesis (p=0.815) and the recruitment of CD11 antigen-like family member B (CD11b)-positive macrophage (p=0.589) were unchanged. Kaplan–Meier survival analysis revealed a better graft survival rate in the vascularized recipient beds pre-treated by *NP2* amiRNA in comparison to controls (p=0.014).

**Conclusions:**

Knockdown of *NP2* improves corneal graft survival by selectively inhibiting lymphangiogenesis in vascularized beds before transplantation. Thus our results open new treatment options for transplant rejection and other lymphatic disorders.

## Introduction

Currently corneal transplantation is the only treatment for many severe cornea diseases including corneal injury, infection, degeneration and inherited diseases. The 5-year survival rate of low-risk keratoplasty (with a preoperatively avascular recipient bed) is around 90%, even without human leukocyte antigen matching [1]. In contrast, the survival rate of high-risk keratoplasty (with a pathologically prevascularized corneal bed) decreases significantly to below 50% due to immune-mediated rejection [2,3]. Preexisting corneal blood (hemangiogenesis) and lymphatic (lymphangiogenesis) vessels in recipient beds have been identified as strong risk factors for immune rejection following corneal transplantation [3,4]. The blood vasculature drains oxygen, nutrients, and cells to corneas whereas the lymphatic vessels transport donor-derived antigen-presenting cells and antigenic materials to the draining lymph nodes, thus inducing an immune response against an allogeneic transplant [5]. Recent studies on corneal hemangiogenesis has demonstrated that anti-hemangiogenic strategies may promote graft survival both in the low-risk as well as in the high-risk murine corneal transplantation [6,7]. Nevertheless, several studies suggest that lymphangiogenesis plays an important role in the induction of alloimmunity after organ transplantation [5]. Using the murine model of corneal transplantation, it was shown that afferent corneal lymphatics may be equal, or even more important than efferent corneal blood vessels in modulating allograft rejection [8].

Although endogenous lymphangiogenic inhibitors remain to be discovered, several secreted factors that promote corneal lymphangiogenesis have been identified recently, including members of the vascular endothelial growth factor (VEGF) family [9], fibroblast growth factor-2 [10], angiopoietin [11], platelet derived growth factor–BB [12], hepatocyte growth factor [13], and insulin-like growth factors [14]. Among these corneal lymphangiogenic factors, lymphatic growth factors VEGF-C and its receptor vascular endothelial growth factor receptor (VEGFR)-3 are best studied. VEGFR-3 has been shown to be expressed in corneal epithelium [15] and corneal dendritic cells [16], and *VEGFR-3* expression is upregulated in inflamed corneas [17]. Anti-lymphangiogenic strategies targeting VEGFR-3-mediated signaling specifically inhibit lymphangiogenesis in inflammatory corneal neovascularisation [18] and significantly suppress corneal transplant rejection [17]. VEGF-C, a ligand of VEGFR-3, has been shown to be able to induce lymphatic vessel growth in the cornea [9,10]. In addition, *VEGF-C* is upregulated by proinflammatory cytokines in macrophages, dendritic cells, neutrophils and mast cells [19], suggesting that it stimulates lymphatic vessel growth during inflammation.

Neuropilin-2 (NP2) is a transmembrane protein initially identified as a receptor for class-3 semaphorin subfamily for neuronal guidance [20]. However, NP2 also acts as a co-receptor for VEGF-C [21] and is implicated in embryonic vessel development [22]. More recent studies have revealed that NP2 functions in tumor lymphangiogenesis and tumor metastasis [23]. This raises the intriguing possibility that NP2 may be a modulator of corneal lymphangiogenesis and that interfering with the afferent arm of the immune response by blocking NP2 may reduce the risk of corneal graft rejection.

Therefore, in this study, we employed artificial microRNA (amiRNA) to knockdown *NP2* in lymphatic endothelial cells (LECs) and further achieved selective inhibition of lymphangiogenesis in suture-induced vascularized corneal beds by intracorneal administration of *NP2* amiRNA. Finally we established a mouse model of high-risk orthotopic corneal transplantation to demonstrate that selective inhibition of lymphangiogenesis mediated by *NP2* knockdown led to improved high-risk graft survival on vascularized recipient beds before transplantation.

## Methods

### Construction of plasmid

**Figure 1:**
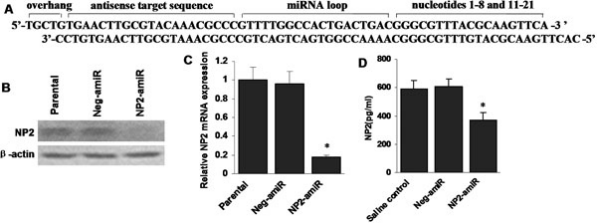
*NP2*-targeting artificial microRNA (amiRNA) down-regulates VEGF-C induced *NP2* expression in mouse LECs. **A**: Double-stranded oligonucleotide encoding a pre-miRNA against mouse *NP2*. **B**: Mouse LECs were transfected with *NP2*-targeting amiRNA (NP2-amiR), negative amiRNA (neg-amiR) or mock-transfected (parental), 48 h after transfection *NP2* expression was detected by western blot using anti-NP2 antibody. The images were representative of three independent experiments. β-actin was used as loading control. **C**: Quantitative real-time PCR analysis of *NP2* mRNA in parental, negative amiRNA or *NP2* knockdown cells. Data were presented as the mean transcript expression normalized to *GAPDH* of three independent samples±SEM (n=3 per condition). The asterisk indicates a p<0.01 versus parental control. **D**: ELISA analysis of NP2 level in mouse corneas at the time point (7 days after injury; n=9 per group). All measurements were performed in duplicate. Bars, SEM. The asterisk indicates a p<0.05 versus saline control.

Using the Invitrogen’s RNAi web design tool, we designed a potential mouse *NP2*-specific targeting sequence as described previously [24]. A BLAST analysis was performed to ensure the designed sequence would have no substantial homology to sequences in other vertebrate genes. The sequence of oligonucleotides was shown in Figure 1A. These oligonucleotides were annealed and ligated into the pcDNA^TM^6.2-GW/EmGFP-miR vector (Invitrogen, Carlsbad, CA) to construct *NP2*-targeting amiRNA (NP2-amiR). The negative control plasmid, negative amiRNA (neg-amiR; Invitrogen), was created using a sequence predicted not to target any known vertebrate genes.

### Cell culture and transfection

The mouse LECs were kindly provided by Dr. Annunciata Vecchi (Istituto Clinico Humanitas, Rozzano, Italy) and cultured as described previously [25]. Cells at passages 14–16 were selected for further experiments. One day before transfection, 1.5×10^5^ cells were seeded into 6-well plates. The cells were then transfected with NP2-amiR or neg-amiR using Lipofectamine PLUS reagent (Invitrogen) and selected with 5 ug/ml blasticidin (Invitrogen). The mouse LECs were then stimulated by 200 ng/ml VEGF-C (Genentech, South San Francisco, CA), and quantitative real-time PCR and western blot were performed 6 h after stimulation.

### Quantitative real-time PCR

Total RNA was extracted using TRIzol reagent (Invitrogen) according to the manufacturer’s instruction. Total RNA (500 ng) was reverse transcribed for 45 min at 45 °C using TaKaRa RNA PCR Kit (AMV) Ver.3.0 (TaKaRa, Dalian, China). EvaGreen quantitative PCR was performed in an ABI Prism 7000 Sequence Detection System (Applied Biosystems, Foster City, CA). Synthesized cDNA (1 μl) of each reaction was used in a 25 μl reaction volume containing 1.25 ul of EvaGreenTM dye (Biotium, Hayward, CA). Results were derived from the comparative threshold cycle method and normalized by glyceraldehyde-3-phosphate dehydrogenase (*GAPDH*) as an internal control. The following primers were used for real-time PCR: *NP2*, 5′-GTG TAC GAC CAT GCC AAG TG-3′ (sense), 5′-TGA CCC AAA GGA GTT TGC TT-3′ (antisense); *GAPDH*, 5′-GTA TT GGG CGC CTG GTC ACC-3′ (sense), 5′-CGC TCC TGG AAG ATG GTG ATG G-3′ (antisense).

### Western blot analysis

Total protein extracts were prepared by washing cells with phosphate-buffered saline (PBS) and lysing in ice-cold RIPA lysis buffer (Beyotime, Shanghai, China) with the addition of protease inhibitors. After separation by 10% sodium dodecyl sulfate-PAGE, proteins were electro-transferred onto nitrocellulose membranes. The membranes were incubated in blocking solution (2% BSA in Tris-buffered saline with Tween-20) for 1 h at room temperature, then incubated with rabbit polyclonal anti-NP2 antibody (Santa Cruz Biotechnology, Inc., Santa Cruz, CA) overnight at 4 °C. After washing and blocking, the membranes were further incubated with corresponding horseradish peroxidase-conjugated second antibody for 1 h at room temperature, and developed using enhanced chemiluminescence detection reagents (Applygen Technologies Inc., Beijing, China). Quantitative analysis of the bands was performed with LabWorks^TM^ Image Acquisition and Analysis Software (GDS-8000 system; UVP, Cambridge, UK).

### Animals and anesthesia

Six- to eight-week-old male BALB/c and C57BL6 mice were purchased from the Institute of Tumor Research (Harbin, China). Animals were treated in accordance with the ARVO Statement for the Use of Animals in Ophthalmic and Vision Research. Mice were anesthetized with a mixture of ketamine and xylazine (120 mg/kg bodyweight and 20 mg/kg bodyweight, respectively).

### Suture-induced corneal neovascularization and corneal intrastromal injections

Inflammatory corneal neovascularization was induced by corneal suture placement, as described previously [26] with some modifications. Briefly, a 2-mm corneal trephine was gently placed on the cornea to mark the central corneal area. Eight interrupted 11–0 nylon sutures (Huawei, Hangzhou, China) were placed in the site where the corneal trephine marked. Corneal sutures were removed after 7 days. The in vivo delivery of NP2-amiR or neg-amiR to the cornea was performed as described previously [27]. Briefly, 1.0 ug plasmid in 2 ul of PBS was injected into the corneal stroma using a 33-gauge Hamilton syringe (Hamilton Co., Reno, NV). Injections were performed on day 3 after suture placement. One week later, penetrating corneal transplantations were performed using age-matched C57BL/6 donors.

### High-risk corneal transplantation and the evaluation of graft survival

Mouse orthotopic corneal penetrating keratoplasty was performed as described previously [4]. Briefly, the central corneal area of C57BL6 donor was excised using a 2.0-mm mouse trephine and Vannas scissiors. The prevascularized graft bed of BALB/c recipient was also prepared by trephining the right eye and the donor graft was sewn into place using 8 interrupted 11–0 nylon sutures (Sharppoint; Vanguard, Houston, Tex). Corneal transplant sutures were removed after 7 days and grafts were then examined by slitlamp microscopy twice weekly over 56 days. All grafted eyes with surgical complications (hyphema, cataract, infection, significant anterior synechiae, or loss of anterior chamber) were excluded from the study. The degree of corneal grafts for opacity was graded and the survival rates were assessed by Kaplan–Meier analysis [28]. Grafts with an opacity score of 2 or higher after 2 weeks or an opacity score of 3 or higher at 2 weeks were regarded as rejected.

### Immunohistochemistry

Corneal whole mount preparations were done as previously described [7]. Briefly, corneas were dissected from the eye behind the corneal limbus, rinsed in PBS and fixed in acetone for 1 h at room temperature. Then the corneas were washed in PBS and blocked in 2% BSA in PBS for 2 h at room temperature. Afterward, the corneas were incubated with rabbit anti-mouse LYVE-1 antibody (1:500; Abcam, Cambridge, UK) overnight at 4 °C. On the 2nd day, the tissue was washed, blocked and stained with FITC-conjugated rat anti-mouse CD31 antibody (1:200; Santa Cruz Biotechnology, Santa Cruz, CA) overnight at 4 °C. On the 3rd day, the specimens were incubated with CY3-conjugated goat anti-rabbit second antibody (1:100; Sigma, St. Louis, Mo.) for 1 h at room temperature. To detect the recruitment of macrophages into the inflamed cornea, FITC-conjugated rat anti-mouse CD11b antibody (BD PharMingen, San Diego, CA) was used. To ensure specificity, negative controls were performed by omitting the primary antibody. Fluorescence microscopy and photography was done using the BX51 camera (Olympus Optical Co., Hamburg, Germany). The immunofluorescence was quantified using NIH Image software.

### Determination of NP2 levels by ELISA

Corneas harvested for ELISA were homogenized in 1.0 ml of sterile PBS containing 0.05% v/v Triton-X 100 at 4 °C. The supernatant was collected, and total protein was determined with a Bradford protein assay. The level of NP2 in corneal lysates was determined by ELISA kit for murine NP2 (R&D systems, Minneapolis, MN) according to the manufacturer’s instructions. All measurements were performed in duplicate.

### Statistical analysis

All statistical analyses were performed using SPSS 13.0 software (SPSS, Chicago, IL). The differences between groups were compared by using Student’s *t*-test for all in vitro studies and the Mann–Whitney U test for all in vivo studies. A p<0.05 was considered significant.

## Results

### *NP2*-targeting amiRNA suppresses VEGF-C-induced *NP2* expression in mouse LECs

Western blot assay demonstrated that NP2-amiR but not negative control neg-amiR specially inhibited *NP2* expression in mouse LECs stimulated by VEGF-C (Figure 1B). Furthermore, real-time quantitative PCR assay confirmed the significant inhibition of *NP2* mRNA expression in NP2-amiR treated cells compared with parental control cells (p*=*0.005, Figure 1C).

### Intrastromal delivery of *NP2*-targeting amiRNA suppresses corneal NP2 expression

To further evaluate whether NP2-amiR could inhibit *NP2* expression in vivo, corneas were subjected to intrastromal delivery of NP2-amiR and harvested on day 7 after suture placement. NP2 ELISA showed that NP2 concentration was 591.14±57.51 pg/ml in saline control mice, 605.58±52.58 pg/ml in neg-amiR treated mice (p=0.606, compared to control), and 372.62±50.89 pg/ml in mice injected with NP2-amiR (p=0.031, compared to control, Figure 1D). These data confirmed that the intrastromal delivery of NP2-amiR resulted in reduced *NP2* expression.

### Intrastromal delivery of *NP2*-targeting amiRNA inhibits corneal lymphangiogenesis

**Figure 2:**
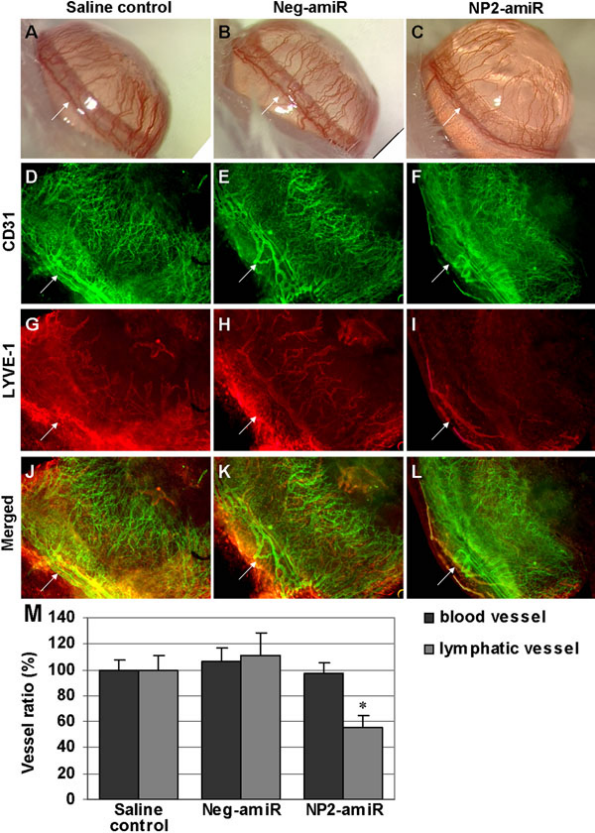
Intrastromal injection of *NP2*-targeting amiRNA inhibits suture-induced corneal lymphangiogenesis but not hemangiogenesis. **A**-**C**: Representative images showing that corneal suture injury induced a robust neovascular response seven days after injury. **D**-**L**: Representative segments of corneal whole-mounts (blood vessels in green: CD31/FITC; lymphatic vessels in red: LYVE-1/Cy3) in eyes treated with NP2-amiR (F, **I**, **L**, n=9 mice) or neg-amiR (**E**, **H**, **K**, n=7 mice) compared with the saline control eyes (**D**, **G**, **J**, n=8 mice). Arrows: limbus. Original magnification, 100×. **M**: Quantification of immunohistochemical staining in the area of CD31-possitive blood vessels and LYVE-1-possitive lymphatic vessels. Bars, SEM. The asterisk indicates a p<0.05 versus saline control. The vasculized area of control group was defined as 100%.

Corneal suture placement induced a robust neovascular response which emerged from day 3 and reached suture placement sites at day 7 after suture injury (Figure 2A-C). We investigated the effect of intrastromal injection of *NP2*-amiR on the outgrowth of blood and lymphatic vessels in the suture-induced corneal neovascularisation assay. The densities of Platelet Endothelial Cell Adhesion Molecule-1 (CD31)-positive blood vessels and lymphatic vessel endothelial receptor-1 (LYVE-1)-positive lymphatic vessels were detected by immunohistochemistry on day 7 (Figure 2D-L). Quantitative immunohistochemical and morphometric analyses revealed that the number of lymphatic vessels in mice treated with NP2-amiR was significantly decreased (p=0.015), whereas the number of blood vessels showed no changes, in comparison with saline control (p=0.815, Figure 2M).

### Intrastromal delivery of *NP2*-targeting amiRNA has no effect on cell infiltration

**Figure 3:**
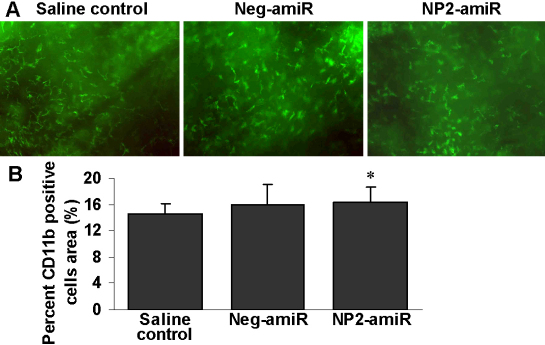
Intrastromal injection of *NP2*-targeting amiRNA has no effect on the recruitment of macrophage into the cornea. **A**: Immunohistochemical staining for CD11b in corneal whole-mounts (n=6 mice per group). Original magnification, 200×. **B**: Quantitative analysis of immunohistochemistry showed that *NP2* knockdown had no significant effect on the recruitment of CD11b-positive macrophages into the cornea immediately before high-risk corneal transplantation (7 days after suture placement). Bars, SEM. The asterisk indicates a p>0.05 versus saline control.

To determine the effect of reduced *NP2* expression on cell infiltration associated with corneal suture injury in recipient beds immediately before transplantation, we next examined CD11 antigen-like family member B (CD11b)-positive macrophage infiltration into inflamed corneas in mice treated with NP2-amiR. As shown in Figure 3, there was no significant difference in the number of inflammatory cells between mice treated with NP2-amiR and control mice (p=0.589).

### Intrastromal delivery of *NP2*-targeting amiRNA in the recipient bed before transplantation significantly promotes subsequent graft survival

**Figure 4:**
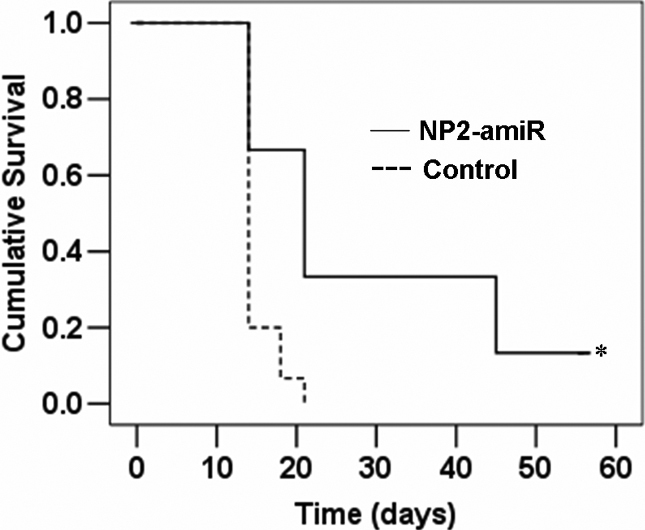
Effect of *NP2* inhibition by NP2-amiR on survival of high-risk allogeneic corneal transplants. Kaplan–Meier analysis showing significant improvement of corneal graft survival in BALB/c corneal beds pretreated with NP2-amiR (n=13) before transplantation compared with beds pretreated with saline solution (n=15). The asterisk indicates a p<0.05 versus saline control.

The fact that afferent corneal lymphatics plays a crucial role in allograft rejection [8], together with our data that blockade of *NP2* selectively inhibited corneal lymphangiogenesis, prompted us to investigate whether knockdown of *NP2* could improve corneal allograft survival. Results from Kaplan–Meier survival curves showed significantly prolonged graft survival in the recipient beds pretreated with NP2-amiR, relative to control beds injected with saline solution (p*=*0.014, Figure 4).

## Discussion

Increasing evidence suggests that NP2, a coreceptor for VEGFC, emerges as a potent regulator of lymphangiogenesis in embryonic vessel development, tumor lymphangiogenesis and metastasis. However, the effect of *NP2* blockade on immunomodulation following transplantation has not been investigated. To our knowledge, our findings for the first time demonstrate that RNA interference-mediated blockade of *NP2* is a potential therapeutic avenue to improve corneal allograft survival by selective inhibition of lymphangiogenesis on vascularized recipient beds before transplantation.

Afferent lymphatic vessels (afferent arm of the immune reflex arc), efferent blood vessels (efferent arm of the immune reflex arc) and antigen-presenting cells such as macrophages play major roles in the process of immunomodulation after corneal transplantation. However, in severe corneal inflammatory disease, corneal lymphatic vessels and blood vessels often grow in parallel into the cornea [29], which makes it difficult to separate lymphangiogenesis from hemangiogenesis. Whether hemangiogenesis and lymphangiogenesis contribute equally to corneal transplant rejection has not been well studied, largely due to the difficulty in selectively dissecting the individual contributions of these two closely intermingled components. Our results suggest *NP2* knockdown as an effective strategy for specific inhibition of lymphangiogenesis without obvious effects on hemangiogenesis. Furthermore, *NP2* is not expressed in corneal macrophage (data not shown), and *NP2* knockdown had no apparent effect on the macrophages recruitment into vascularized beds at the time of corneal transplantation. These data demonstrate that decreasing *NP2* expression has no direct effect on corneal macrophages trafficking to regional lymph nodes, and further confirm the selective effect of *NP2* knockdown in the inflamed cornea. In the aggregate, our findings indicate that *NP2*-targeting amiRNA may represent a novel tool to investigate the individual properties of hem- or lymphangiogenesis. More importantly, our results indicate that inhibition of *NP2* provides novel treatment options for disorders associated with pathological lymphangiogenesis without causing the corresponding complications induced by anti-hemangiogenic effect.

In addition, our data showed that selective inhibition of lymphangiogenesis on vascularized recipient beds before transplantation by blockade of *NP2* led to significantly improved graft survival. Afferent lymphatic vessels act as a conduit through which antigen-presenting cells and soluble antigenic materials migrate to the draining lymph nodes to induce an immune rejection [5]. Suppression of lymphangiogenesis in the graft bed by *NP2* knockdown may reduce the opportunity of donor-derived immune cells to enter the lymphatic vascular system, thus mitigating the immune response against an allogeneic transplant and leading to better graft survival. This is in line with the finding that the absence of lymphatic vessels before transplantation significantly promoted subsequent graft survival using VEGFR-3 Abs or anti-integrin a5 small molecules [30]. These observations, together with our current findings, led us to postulate that corneal allograft survival can be significantly improved by the reduction of lymphatic vessels in the recipient bed alone. Interestingly, NP2 is actively involved in the regulation of lymphatic vasculature by modulating VEGFR-3 signaling [31], and blocking VEGFR-3 decreases corneal dendritic cell trafficking to regional lymph nodes in a mouse corneal transplantation model [17]. Thus we speculate that loss of NP2 promotes allograft survival at least in part by inhibiting dendritic cell trafficking through VEGFR-3-mediated signaling.

Besides the new therapeutic use for improving corneal graft survival, our data also reveal *NP2*-targeting amiRNA as a promising treatment in solid organ transplantation, as it does not affect the blood supply, which is essential for wound healing, nutrition, and homeostasis of the graft. Furthermore, selective anti-lymphangiogenic therapy might be an optional strategy in other diseases such as lymphatic vascular malformations, lymphatic neoplasms, tumor lymphangiogenesis and lymphatic metastases.

Collectively, our results demonstrate that intracorneal administration of *NP2*-targeting amiRNA selectively inhibited lymphangiogenesis without affecting hemangiogenesis and macrophages recruitment, and Kaplan–Meier survival analysis revealed a better graft survival rate in the *NP2*-targeting amiRNA treated vascularized corneal beds before transplantation. These data open new treatment options for transplant rejection and other lymphatic disorders.
